# iTRAQ-based proteomics profiling of Schwann cells before and after peripheral nerve injury

**DOI:** 10.22038/IJBMS.2018.26944.6588

**Published:** 2018-08

**Authors:** Gui-Dong Shi, Xin Cheng, Xian-Hu Zhou, Bao-You Fan, Yi-Ming Ren, Wei Lin, Xiao-Lei Zhang, Shen Liu, Yan Hao, Zhi-Jian Wei, Shi-Qing Feng

**Affiliations:** 1 Department of Orthopedics, Tianjin Medical University General Hospital, 154 Anshan Road, Heping District, Tianjin, China; 2 Tianjin Neurological Institute, Key Laboratory of Post-Neuroinjury Neuro-repair and Regeneration in Central Nervous System, Ministry of Education and Tianjin City, Tianjin, China

**Keywords:** Biomarkers, Peripheral nerve injury, Proteomics, Proteins, Schwann cells

## Abstract

**Objective(s):**

Schwann cells (SCs) have a wide range of applications as seed cells in the treatment of nerve injury during transplantation. However, there has been no report yet on kinds of proteomics changes that occur in Schwann cells before and after peripheral nerve injury.

**Materials and Methods:**

Activated Schwann cells (ASCs) and normal Schwann cells (NSCs) were obtained from adult Wistar rat sciatic nerves. After immunofluorescence identification, we identified differentially expressed proteins in the ASCs and NSCs using isobaric tags for relative and absolute quantitation (iTRAQ) combined with high-resolution Orbitrap liquid chromatography-mass spectrometry/mass spectrometry (LC-MS/MS). In addition, all the differentially expressed proteins were analyzed by Gene ontology (GO) analysis and Kyoto encyclopedia of genes and genomes (KEGG) pathway analysis. Finally, several differentially expressed proteins were selected for Western blot verification.

**Results:**

A total of 122 differentially expressed proteins in ASCs and NSCs were screened. GO analysis suggested that these different proteins are likely to accumulate in the cytoplasm and are associated with single-multicellular organism processes. The KEGG pathway analysis suggested that proteins related to purine metabolism were significantly enriched. The expression of Transmembrane glycoprotein NMB (GPNMB), Ectonucleotide pyrophosphatase/phosphodiesterase family member 3 (ENPP3), and other proteins were consistent with the proteomics data obtained by Western blot analysis.

**Conclusion:**

GPNMB, ENPP3, GFPT2, and other proteins may play an important role in the repair of peripheral nerve injury. This study may provide new insights into changes in SCs after peripheral nerve injury.

## Introduction

With the development of innovative technologies in the medical field worldwide, treatment of nerve injury tends to be diversified. In addition to the central nervous system (CNS) damage, peripheral nerve injury has become a common concern among scientists and doctors around the world. (1-3). Since the previous drug and surgical treatments to the emergence of cell therapies today, treatment technology has been constantly updated, but there is also a corresponding emergence of some problems (4-6). In this study, we assess the differential protein expression of Schwann cells before and after peripheral nerve injury and proceed to explore some of the changes in protein expression occurring in the cell.

Schwann cells have gained increasing attention in the field of nerve regeneration owing to their ability to repair nerve injury and promote axonal regeneration and myelination (7-10). Recently, treatment with SCs combined with other stem cells, such as mesenchymal or neural stem cells, and other treatment strategies have also been more widely recognized (11-14). However, the role of SCs in repair of the peripheral nerves and the underlying specific pathophysiological mechanisms are still unknown. Furthermore, after peripheral nerve injury and Wallerian degeneration (15, 16), changes in the proteomics of SCs have not yet been clearly reported through specific studies.

Isobaric tags for relative and absolute quantitation (iTRAQ) is an equal weight labeling technique for relative and absolute quantification of protein (17-19). This technique allows comparison between proteins in varied samples, such as differences in protein expression levels in tissue samples under different pathological conditions or at different developmental stages (20). After iTRAQ labeling, high-precision mass spectrometer in series analysis can be performed and protein expression of up to 8 samples can be compared (21). Based on the above techniques, we selected SCs before and after peripheral nerve injury for iTRAQ labeling and mass spectrometry.

In summary, we isolated and purified SCs before and after peripheral nerve injury. After extracting the protein from SCs and labeling with iTRAQ, the samples were subjected to mass spectrometry to reveal a differentially expressed protein in the sample. The differentially expressed proteins were subjected to gene ontology (GO) annotation and Kyoto encyclopedia of genes and genomes (KEGG) analyses, and several proteins were identified by Western blotting. This study revealed the changes in SCs before and after peripheral nerve injury and formed a basis for subsequent cell therapy.

## Materials and Methods

***Animals and experimental groups***

Nine Wistar rats (4-week-old, approx. 100±10 g, provided by Radiation Study Institute-Animal Center, Tianjin, China) were used in this study. Sciatic nerve injury surgeries were done as described previously (22). Rats were sacrificed and the sciatic nerves of each Wistar rat were isolated and the SCs were extracted. This study contained two major groups—Group A: ASCs from the ligation of the sciatic nerves and Group B: NSCs from the untreated sciatic nerves. All animal breeding experiments were performed according to the Guidelines for Laboratory Animal Safety and Care as issued by the United States National Institutes of Health. All procedures performed in the study involving animals were consistent with the ethical standards set by the above-mentioned institutions.

***Isolation and culture of normal Schwann cells and activated Schwann cells***

SCs were obtained from the 7-day pre-degenerated sciatic nerve of adult male Wistar rats (n = 9) according to a previous study (23). Briefly, nine adult Wistar rats were anesthetized with 10% chloral hydrate (0.3 ml/100 g). After anesthesia satisfaction, the unilateral sciatic nerve was ligated in each rat. After one week, nine rats were sacrificed and the bilateral sciatic nerves of each rat were isolated. After removal of the epineurium, the nerve was washed three times with PBS and 2% antibiotic solution (penicillin, streptomycin) was added. Next, the remaining nerve tissue was cut into small pieces (0.5–1.0 mm^3^). The nerve tissue was digested in a 2-ml mixture for 10–15 min using an equal volume of 0.25% trypsin (Sigma) and 0.06% collagenase (Sigma) at 37 °C and 5% CO_2_. After washing in DMEM/F-12, the tissue pieces were gently dispersed by pipetting and were centrifuged (300 × g, 5 min) to remove the supernatant. An appropriate amount of DMEM/F-12 medium containing 10% FBS was added and the cells were inoculated in a 25-ml culture flask at 37 ^°^C and 5% CO_2_. After three weeks, the cells reached 90% confluency and were used in this experiment after three passages.

***Immunofluorescence staining of Schwann cells***

Cells were seeded at a density of 30,000 cells/well in a 24 Well Clear TC-Treated Multiple Well Plate. After 72 hr, they were fixed for 30 min in 4% (w/v) paraformaldehyde at room temperature. Then, the cells were washed in phosphate buffered saline before the addition of 5% (v/v) normal donkey serum together with 0.1% Triton X-100 (v/v) in PBS for a further 20 min at room temperature. After the blocking serum was removed, the primary antibodies, rabbit monoclonal anti-S100 (Gibco) at respective dilutions of 1:100 were added and the samples were incubated overnight at 4 °C. The cells were then washed in PBS, FITC conjugated donkey anti-rabbit IgG (1:200 dilution) was added and the samples were incubated for 2 hr at room temperature. After the reaction, the cells were washed three times with PBS, and the DAPI nuclear label (Sigma) was applied for 10 min. The Schwann cells were then examined under a fluorescence microscope (Leica DM2500, Germany).

***Sample preparation and iTRAQ labeling***

The medium was removed and the cells were washed three times with 1× phosphate buffered saline (PBS). 300 μl lysis buffer (10% SDS and TEAB) was added to the mixed sample and subjected to tissue homogenization and sonication on ice. After centrifugation at 17,000 × g for 10 min at 4 ^°^C, the supernatant was collected and transferred to a new tube. The obtained protein extract was quantitated by BCA assay (Transgene Biotech) following the manufacturer’s protocol. To ensure that the data are available for technical and biological duplication, each group includes at least 3 repeated protein extracts. The final volume of the protein mixture was adjusted to 300 μl with 100 mM TEAB (triethylammonium bicarbonate, Santa Cruz, USA).

The extracted protein was labeled with a lightly modified iTRAQ® reagent (AB Sciex Inc., MA, USA) according to the manufacturer’s instructions. Each sample was labeled with an isobaric tag for 3 hr at room temperature as follows: the proteins from ASCs were labeled with iTRAQ reagents 127,129, and 131 and those from NSCs were labeled with iTRAQ reagents 126,128, and 130. Finally, all samples were pooled before being subjected to separation techniques and analysis by tandem mass spectrometry.

***Orbitrap LC-MS/MS analysis***

For LC-MS/MS analysis, approximately 200 ng of each fraction was injected. Peptides were separated by LC-MS/MS coupled to an LTQ Orbitrap Velos mass spectrometer. At a resolution of 60,000, the MS spectra were acquired on Orbitraps in the range of 300–2000 m/z. The five most intense ions per survey were selected for collision-induced dissociation fragmentation to be analyzed in the linear trap. 

***Data analysis and quantitation***

The masses of the peptide modifying the Tandem Mass Tags (TMT) zero, duplex, and sixplex reagents are present in the UNIMOD database (www.unimod.org Accessed 8 April 2017). Thermo Scientific Proteome Discoverer 1.1 and other software packages directly support the modification of the TMT reagent and the relative quantification of the reporter ions released from the labeled peptide. For data obtained using a combination of segmentation methods, the proteome discoverer may need to combine the spectra used for identification and quantification.

***Bioinformatics analysis***

Proteins/peptide sequences were imported into Cytoscape (version 3.4.0) for GO annotation. The screening of 122 differentially expressed proteins for GO annotation was performed from the biological process, molecular function, and cellular component. The KEGG-GENES corresponding to the differentially expressed proteins were then analyzed by KEGG Orthology (KOs) and were mapped to KEGG pathways. Protein-protein networks that reveal significantly differentially expressed proteins were analyzed using the Cytoscape software.

***Western blotting***

Of the 122 differentially expressed proteins, several proteins were randomly selected for Western blot analysis validation. Briefly, the same amount of protein (20 μg) of each sample was loaded on 10% sodium dodecyl sulfate-polyacrylamide gel (SDS-PAGE). After blocking with 5% bovine serum albumin, the membrane was incubated with GPNMB (Anti-GPNMB antibody, Abcam, ab98856), ENPP3 (Anti-ENPP3 antibody, Abcam, ab190823), GFPT2 (Anti-GFPT2 antibody, Abcam, ab190966), and SDPR (Anti-SDPR antibody, Abcam, ab113876), and then incubated with the secondary antibody (1:5000 dilutions, Transgene Biotech). Detection of protein bands was performed using the ECL assay kit. Protein quantification was analyzed using the Image-Pro Plus (version 6.0) software. 

***Statistical analysis***

Prism statistical software (Graph Pad v6.01, CA) was employed for data analysis. All data were reported as the mean±standard deviation (SD) in this study. The data were analyzed using one-way analysis of variance (ANOVA). *P*<0.05 was considered as statistically significant. 

## Results

***Culture and identification of Schwann cells***

**Figure 1:**
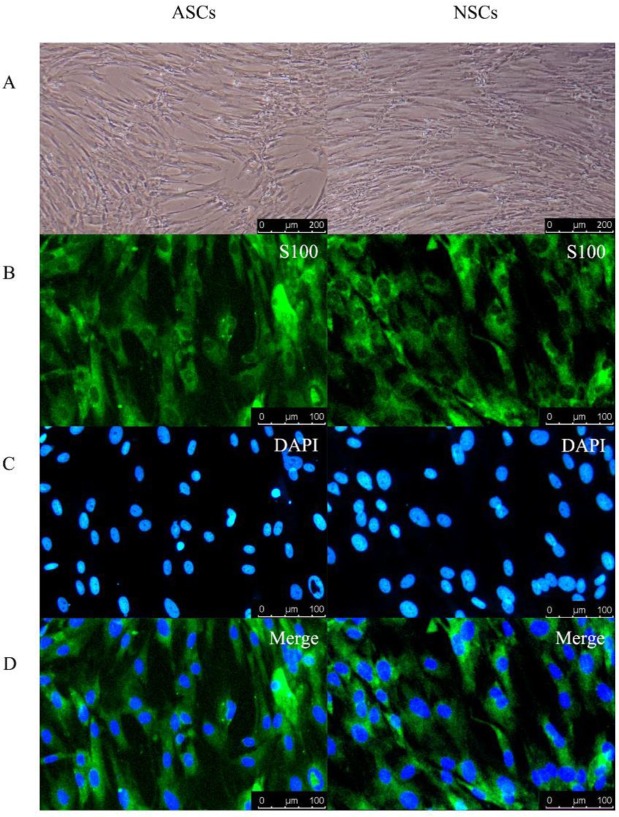
Immunofluorescence staining of Schwann cells. A. The shape of activated Schwann cells (ASCs) and normal Schwann cells (NSCs) under an optical microscope. Both of these SCs, long spindle cells, all were arranged in a fish shape and nuclei were ovoid or oblong. Scale bar: 200 µm. B. SCs were marked with S100 by immunofluorescence. C. The nucleus of SCs was marked with DAPI by immunofluorescence. D. SCs and nuclei of SCs were merged together by immunofluorescence. Scale bar: 100 μm

At 10 days post-isolation of cells, the cells proliferated and covered the entire T75 bottom (Figure 1A). Both the ASCs and NSCs showed positive immunoreactivity for the S100 Schwann cell markers. Figure 1 (B, C, D) shows the expression of these mature markers in Schwann cells, whereas there were no significant differences between these two groups. The specific differences between these two groups can be found in our previous study (24). In conclusion, Schwann cells were prepared for protein extraction after immunofluorescence identification.

***Integrated proteome information***

**Figure 2:**
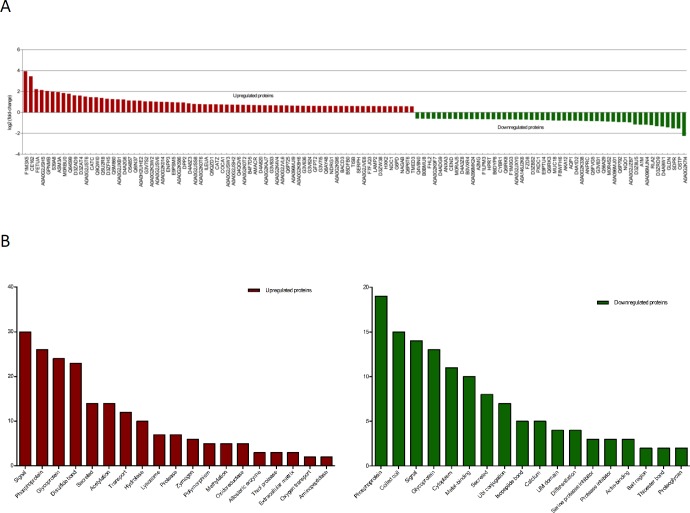
Differentially expressed proteins identified in Schwann cells before and after peripheral nerve injury. A. Proteins that showed increased levels in ASCs are shown in red and those that showed decreased levels are shown in green. B. The upregulated and down-regulated proteins were analyzed by functional clustering

The proteins expressed differentially between NSCs and ASCs were identified by the proteomics approach using iTRAQ. For each sample of SCs before and after peripheral nerve injury, the unique reporter in the low mass region of the MS/MS spectrum was used to measure the relative protein expression level during peptide fragmentation. Eventually, 4473 proteins were identified to be differentially expressed in groups A and B. The screening of differential protein and the selection of fold change were according to previous research (25). A total of 122 proteins were identified (fold ≥ 1.5, *P*-value ≤ 0.05) to be differentially regulated, of which 72 were upregulated (Table 1) and 50 were down-regulated (Table 2). Figure 2A shows the level of up-regulated and down-regulated proteins in ASCs. Moreover, we performed functional clustering analysis of upregulated and down-regulated proteins (Figure 2B).

***GO annotation of differentially expressed proteins***

**Figure 3:**
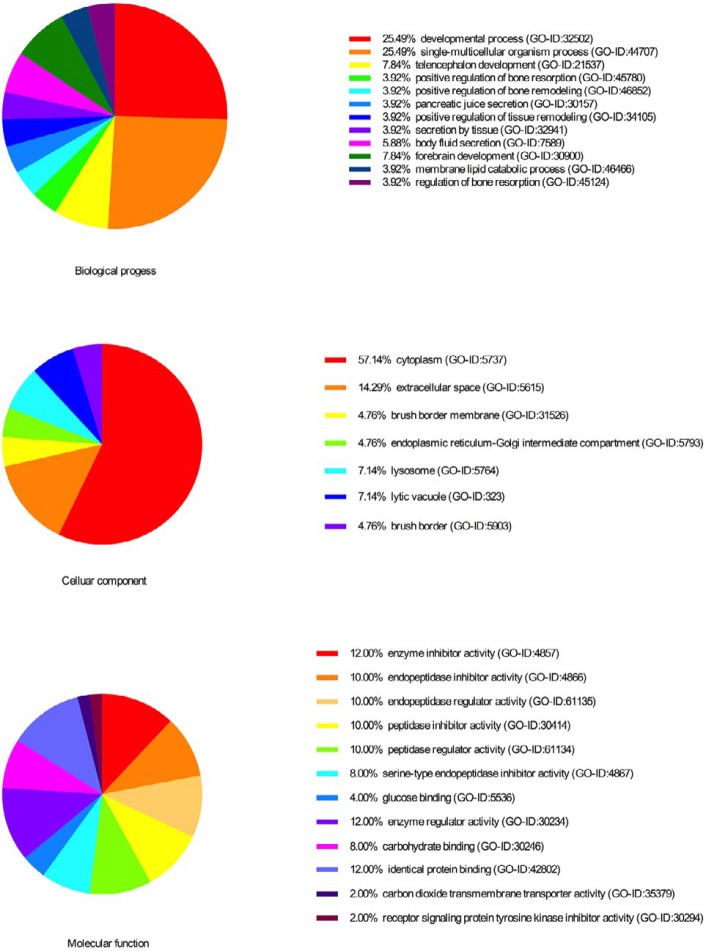
Gene ontology annotation of differentially expressed proteins. Most of the proteins of differential abundance analyzed for the biological process, molecular function, and cellular component were single-multicellular organism process, protein binding, and cytoplasm, respectively

The above differential proteins were further analyzed by Cytoscape (version 3.4.0) software, divided into ‘Molecular function’, ‘Cellular component’, and ‘Biological process’ subcategories (Figure 3).

A biological process is a series of events resulting from an orderly combination of one or more molecules. Of the 122 differentially expressed proteins that were analyzed, most of the proteins were found to be enriched in the single-multicellular organism process (GO-ID:44707) and developmental process (GO-ID:32502). In addition, 57.14% differential expression proteins were located in the cytoplasm (GO-ID:5737), followed by the extracellular space (GO-ID:5615). In the GO annotation analysis, ‘Molecular function’ can provide the function of the gene at the molecular level. In the differentially expressed proteins that were screened, it was found that most of the differential proteins were enriched in enzyme inhibitor activity (GO-ID:4857), followed by identical protein binding (GO-ID:42802), and peptidase regulator activity (GO-ID:61134). Of the 122 differentially expressed proteins, 53 most relevant proteins were screened out and a protein-protein interaction (PPI) network was prepared (Figure 5). In this PPI network, several proteins were further selected for analysis. Moreover, several of these proteins (Thbs2, Lgals3, Cathepsin D (Ctsd), and Sptan1) were chosen for analysis.

***KEGG analysis***

**Figure 4:**
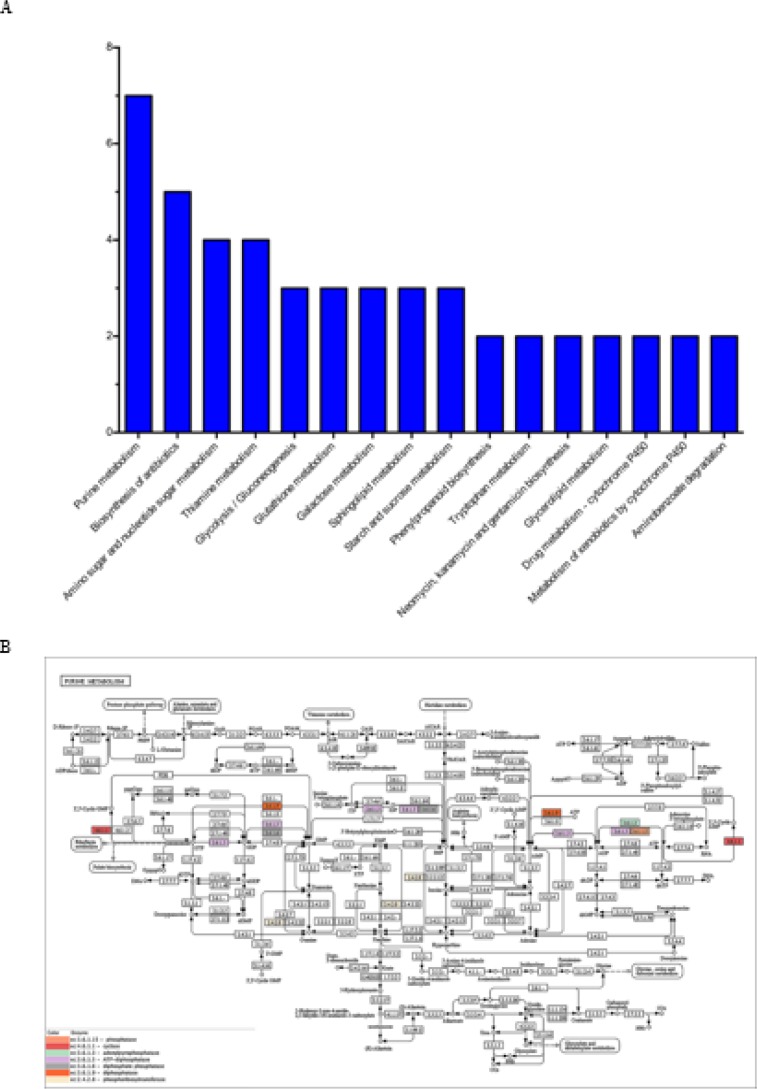
Kyoto encyclopedia of genes and genomes pathway analysis of differentially expressed proteins A. The purine metabolism pathway was enriched in the majority of the differentially expressed proteins. The vertical bars represent the number of the differentially expressed proteins. B. Kyoto encyclopedia of genes and genomes (KEGG) pathway enrichment analysis maps of the Purine metabolism pathway. The proteins in different color frames are differentially expressed proteins identified in this study. The box represents proteins; the arrow represents activation

Protein expression data were mapped to KEGG Mapper-Search & Colour Pathway (http://www.kegg.jp/ Accessed 10 April 2017) to further analyze changes in biological processes. In addition, we counted the most meaningful 16 KEGG pathways, including purine metabolism, biosynthesis of antibiotics, amino sugar and nucleotide sugar metabolism, and Thiamine metabolism. (Figure 4A). In addition, one of the pathways, ‘Purine metabolism’, was chosen for analysis, in which different colors represent different enzymes (Figure 4B). 

***Protein verification by Western blot analysis***

GPNMB, ENPP3, GFPT2, and SDPR were selected in ASC (Group A) and SC (Group B) samples by Western blot analysis. The change in protein abundance as detected in Western blot analysis and protein quantification was highly consistent with that in the proteomics data of SCs (Figures 6A and 6B).

## Discussion

In this study, the proteomics of SCs before and after peripheral nerve injury were studied by using iTRAQ and high-resolution Orbitrap LC-MS/MS. Compared with that in NSCs, 122 differentially expressed proteins were identified in ASCs, of which 72 were upregulated and 50 were down-regulated. In addition, several proteins were randomly selected for Western blot analysis, and the results were consistent with those of proteomics analysis.

**Figure 5:**
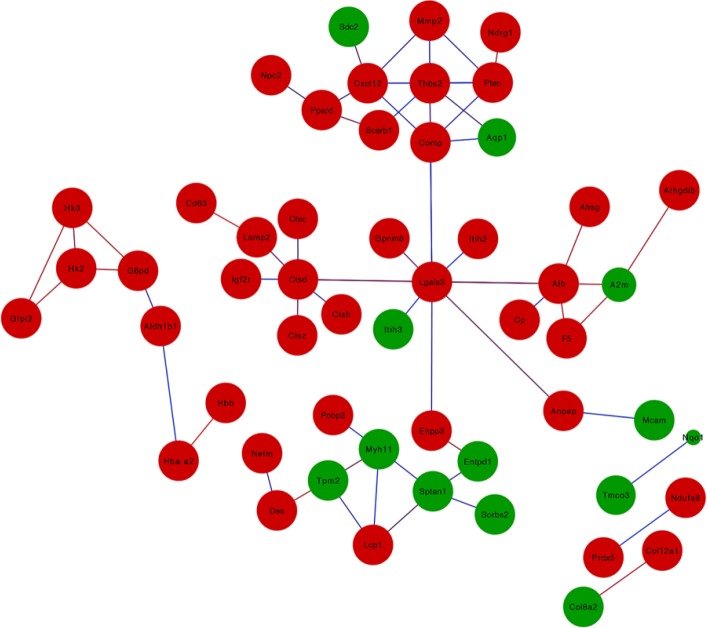
The protein-protein interaction network of significantly differentially expressed proteins was analyzed by the Cytoscape software. Proteins that showed increased levels in ASCs are shown in red and those that showed decreased levels are shown in green. The size of the node shows the significance of the *P*-value, the smaller the *P*-value, the larger the diameter of the node. The color of the edge shows the correlation between the nodes, the red indicates high correlation, and blue indicates low correlation

**Figure 6:**
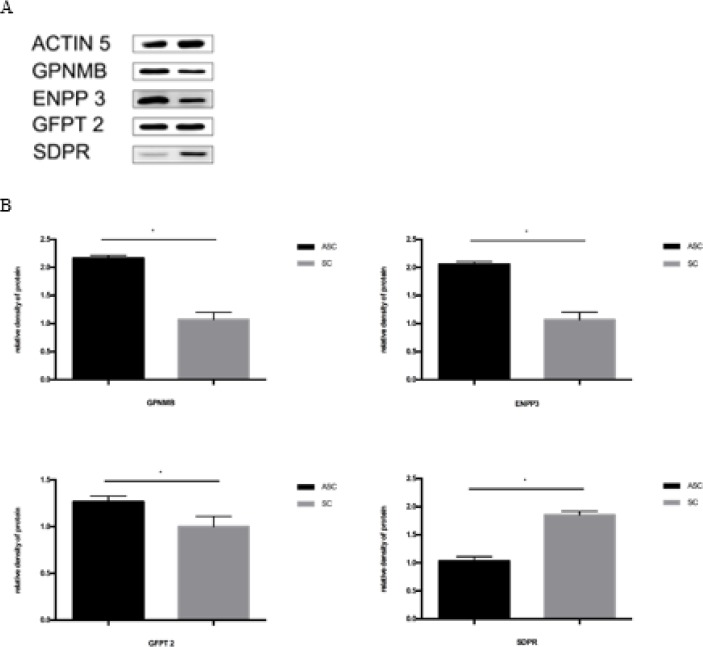
Western blotting analysis of 4 differentially expressed proteins A. ACTIN 5 was used as a loading control for both ASCs and SCs. B. Changes in protein abundance shown by Western blotting analysis and quantification of the proteins was highly consistent with that shown in the proteomics data of SCs (*P<0.05)

**Table 1.** The differentially expressed proteins between normal Schwann cells and activated Schwann cells (up-regulated)

Accession	Protein names	t-test *P-*Value	Fold change
F1M3X5	Maestro heat-like repeat family member 6	6.33156E-25	15.38461538
CE162	Centrosomal protein of 162 kDa	9.00208E-28	10.98901099
FETUA	Alpha-2-HS-glycoprotein	1.7734E-19	4.694835681
A0A0G2JSH5	Serum albumin	9.65752E-21	4.424778761
GPNMB	Transmembrane glycoprotein NMB	4.86769E-20	4.166666667
S39A8	Zinc transporter ZIP8	4.54253E-20	3.95256917
ASM3A	Acid sphingomyelinase-like phosphodiesterase 3a	4.61478E-22	3.861003861
M0RBU0	Cartilage oligomeric matrix protein	7.40253E-19	3.636363636
Q64599	Hemiferrin	1.69731E-19	3.460207612
D3ZAD9	NLR family, pyrin domain-containing 9	6.03881E-19	3.105590062
D3ZAT4	Serine (Or cysteine) peptidase inhibitor, clade A , member 9	5.67045E-20	3.067484663
A0A0G2JST6	Hexokinase 3, isoform CRA_a	9.14012E-22	2.873563218
CATC	Dipeptidyl peptidase 1	6.28748E-16	2.747252747
Q9QWI0	Peroxisome proliferator activated receptor delta	7.09658E-17	2.739726027
Q5U2R8	Interferon activated gene 204	9.99338E-20	2.617801047
D3ZFH5	Uncharacterized protein	8.84154E-19	2.487562189
Q5M860	Rho GDP dissociation inhibitor beta	1.95139E-13	2.427184466
A0A0G2JXB1	Galectin	1.96743E-17	2.392344498
D4A2G6	Thrombospondin 2	2.19056E-17	2.364066194
O54857	Phosphatase and tensin homolog	6.1274E-17	2.207505519
Q6IN37	GM2 ganglioside activator	6.84766E-17	2.197802198
A0A0H2UHE2	Interleukin 1 receptor antagonist, isoform CRA_c	2.03534E-09	2.183406114
G3V7S2	Neurofilament medium polypeptide	1.33839E-11	2.083333333
A0A0G2K3W2	Coagulation factor V	1.42405E-11	2.083333333
A0A0G2JSV6	Globin c2	4.13128E-19	2.044989775
A0A0G2K014	Lymphocyte cytosolic protein 1	5.41354E-17	2.024291498
ENPP3	Ectonucleotide pyrophosphatase/phosphodiesterase family member 3	1.23695E-13	2.016129032
E9PSM5	72 kDa type IV collagenase	2.08592E-17	1.976284585
A0A0G2K586	Fatty acid-binding protein, adipocyte	1.81024E-12	1.949317739
DPP2	Dipeptidyl peptidase 2	1.1589E-13	1.937984496
D4A8Z3	Ferric-chelate reductase 1	2.47541E-17	1.831501832
A0A0G2JSS8	Peroxiredoxin 5, isoform CRA_c	2.90578E-16	1.754385965
A0A0G2K0T6	Gamma-synuclein	2.426E-10	1.745200698
ILEUA	Leukocyte elastase inhibitor A	7.29657E-12	1.715265866
Q9QZD1	Chemokine (C-X-C motif) ligand 12	4.49991E-09	1.715265866
CATZ	Cathepsin Z	6.04105E-09	1.712328767
COCA1	Collagen alpha-1(XII) chain	3.7569E-07	1.703577513
A0A0G2JSW3	Globin a4	9.84124E-16	1.686340641
A0A0G2JSH2	3-hydroxybutyrate dehydrogenase, type 1, isoform CRA_a	8.2164E-16	1.675041876
Q4QQV6	Lymphocyte specific 1, isoform CRA_a	4.36495E-15	1.672240803
A0A0G2K0T2	Tetraspanin	2.29439E-10	1.661129568
B4F7D5	RGD1566403 protein	2.37434E-14	1.650165017
AMACR	Alpha-methylacyl-CoA racemase	6.32779E-12	1.63132137
D4A820	Cytochrome P450, family 2, subfamily s, polypeptide 1	6.68064E-10	1.618122977
A0A0G2KAJ7	Collagen alpha-1(XII) chain	4.8802E-14	1.607717042
G3V833	Frizzled homolog 1	3.217E-09	1.605136437
A0A0G2K4V4	3-ketodihydrosphingosine reductase	3.44896E-16	1.602564103
A0A0G2JVL6	NADH dehydrogenase [ubiquinone] 1 alpha subcomplex subunit 8	9.34868E-11	1.597444089
Q6P725	Desmin	4.00882E-12	1.594896332
A0A096MJI9	Sodium/potassium-transporting ATPase subunit beta	2.12522E-18	1.57480315
A0A0G2K8H6	Pro-cathepsin H	9.77848E-07	1.564945227
G3V636	Scavenger receptor class B member 1	1.64462E-09	1.557632399
G3V824	Insulin-like growth factor 2 receptor	8.57422E-17	1.552795031
GFPT2	Glutamine--fructose-6-phosphate aminotransferase [isomerizing] 2	5.26705E-09	1.547987616
G3V7I5	Aldehyde dehydrogenase X, mitochondrial	2.6707E-10	1.545595054
Q6AY48	Poly(RC) binding protein 3	4.40202E-09	1.545595054
NDRG1	Protein NDRG1	2.77589E-13	1.543209877
A0A0G2K9I6	Ceruloplasmin	2.93992E-08	1.543209877
BACD3	BTB/POZ domain-containing adapter for CUL3-mediated RhoA degradation protein 3	5.47666E-12	1.538461538
B5DFB0	Leprecan-like 2 (Predicted), isoform CRA_b	2.14094E-09	1.531393568
TISB	mRNA decay activator protein ZFP36L1	3.11572E-11	1.531393568
SERPH	Serpin H1	9.59235E-11	1.529051988
A0A0G2JVE6	Alanyl (Membrane) aminopeptidase	1.98345E-10	1.529051988
F7FJQ3	NPC intracellular cholesterol transporter 2	1.67938E-06	1.526717557
LAMP2	Lysosome-associated membrane glycoprotein 2	5.37796E-09	1.524390244
D3ZW38	Exosome component 6	4.40769E-11	1.522070015
HXK2	Hexokinase-2	1.7815E-14	1.512859304
NDC1	Nucleoporin NDC1	4.33091E-12	1.512859304
G6PD	Glucose-6-phosphate 1-dehydrogenase	1.64107E-09	1.510574018
NAGAB	Alpha-N-acetylgalactosaminidase	1.04058E-14	1.510574018
Q6P6T6	Cathepsin D	4.86308E-15	1.508295626
TMED3	Transmembrane emp24 domain-containing protein 3	4.46256E-11	1.499250375

UniProt accession numbers that can be found on www.uniprot.org. Accessed 8 April, 2017

Fold change: The quantity changes of protein abundance between the two groups

**Table 2:** The differentially expressed proteins between normal Schwann cells and activated Schwann cells (down-regulated)

Accession	Protein names	t-test *P*-Value	Fold change
Q4V8N0	Lipocalin 7, isoform CRA_a	1.1964E-07	0.664893617
B0BMU8	Musculoskeletal, embryonic nuclear protein 1	2.81912E-05	0.663129973
FHL2	Four and a half LIM domains protein 2	4.20985E-09	0.658761528
A0A0G2K9F7	Zinc finger, MYND-type-containing 8	0.001160328	0.657462196
D4ADG9	Collagen type VIII alpha 2 chain	3.31488E-07	0.656598818
ANXA3	Annexin A3	0.000105632	0.654022237
CEND	Cell cycle exit and neuronal differentiation protein 1	4.91826E-06	0.651465798
M0RAJ5	Proline-rich 14-like	9.66231E-07	0.650195059
D4A3Z8	Transmembrane and coiled-coil domain-containing protein 3	2.57001E-05	0.644329897
B0VXR4	JIP3 protein	2.01593E-05	0.643915003
A0A096MK24	MORC family CW-type zinc finger 4	1.60452E-06	0.643086817
A2MG	Alpha-2-macroglobulin	0.000167324	0.642260758
F1LPM3	Sorbin and SH3 domain-containing protein 2	6.13675E-10	0.641025641
HPRT	Hypoxanthine-guanine phosphoribosyltransferase	3.01248E-12	0.639795266
B6DYP8	Glutathione S-transferase	0.000461151	0.636132316
CYBR1	Cytochrome b reductase 1	0.000313257	0.635324015
Q6IRK8	Spectrin alpha chain, non-erythrocytic 1	1.54133E-07	0.631313131
F1M0G3	Ectonucleoside triphosphate diphosphohydrolase 1	1.96875E-09	0.630914826
A0A0G2JXY0	Uncharacterized protein	2.7026E-05	0.62774639
A0A146J2K6	Lasp-2	5.79703E-08	0.624219725
FZD8	Frizzled-8	3.28025E-06	0.623830318
D3ZBS2	Inter-alpha-trypsin inhibitor heavy chain H3	1.02664E-06	0.612369871
PXDC1	PX domain-containing protein 1	4.33643E-06	0.612369871
E9PTU4	Myosin-11	9.78468E-13	0.601684717
Q6IRK3	Syndecan	2.71733E-06	0.597371565
MUC18	Cell surface glycoprotein MUC18	3.79694E-09	0.586510264
F8WFH6	Protein FAM131B	1.09524E-15	0.582411182
AKA12	A-kinase anchor protein 12	4.54799E-14	0.582072177
AQP1	Aquaporin-1	6.2957E-05	0.580383053
D4A1D2	Rho guanine nucleotide exchange factor 26	1.00711E-07	0.580046404
A0A0G2K338	Four and a half LIM domains protein 1	1.79426E-05	0.574712644
ANPRC	Atrial natriuretic peptide receptor 3	3.79533E-08	0.566572238
Q5FVG5	Similar to tropomyosin 1, embryonic fibroblast-rat, isoform CRA_c	6.48253E-10	0.564334086
G3V831	Max dimerization protein 3	1.43159E-10	0.563697858
Q56A29	Visinin-like 1	1.12729E-06	0.555555556
M0R4S2	Apolipoprotein D	1.48461E-06	0.547345375
A0A096MJ01	LIM domain-binding 3	9.90342E-06	0.543478261
Q6P792	Four and a half LIM domains 1	2.29744E-07	0.529661017
NQO1	NAD(P)H dehydrogenase [quinone] 1	0.000698527	0.529661017
A0A0G2JZB7	Neuron navigator 3	1.46506E-08	0.518134715
D3Z8U5	Metalloendopeptidase	1.03836E-10	0.451875282
A1M	Alpha-1-macroglobulin	8.78141E-06	0.447227191
A0A096MJN4	Septin 4	4.76859E-12	0.44603033
RLA2	60S acidic ribosomal protein P2	1.25097E-08	0.430848772
D3ZRD9	Allograft inflammatory factor 1-like	3.31643E-09	0.40371417
D4A9W1	Coiled-coil domain-containing 88C	3.85007E-10	0.394477318
GLDN	Gliomedin [cleaved into: gliomedin shedded ectodomain]	1.71803E-09	0.373552484
SDPR	Serum deprivation-response protein	3.99342E-13	0.348189415
OSTP	Osteopontin	1.41268E-07	0.345781466
A0A0G2K7I4	RCSD domain containing 1	1.27673E-14	0.211282485

UniProt accession numbers that can be found on www.uniprot.org. Accessed 8 April, 2017

Fold change: The quantity changes of protein abundance between the two groups

A total of 122 differentially expressed proteins were obtained from protein mass spectrometry. GO annotation analysis was performed using ‘Molecular function’, ‘Cellular component’, and ‘Biological process’. GO annotation analysis results show that these different proteins are likely to accumulate in the cytoplasm and are associated with single-multicellular organism processes. Moreover, we found that pyruvate metabolism, biosynthesis of antibiotics, and amino sugar and nucleotide sugar metabolism pathways were significantly enriched in the KEGG pathway analysis. In the PPI network, Thbs2, Lgals3, Ctsd, and Sptan1 are four proteins related to the development of the CNS and peripheral nervous system (26-30). Previous research reported that Thbs2 can promote axonal regeneration and synaptic formation (31). Researchers found that Sprague-Dawley pregnant rats exposed to drinking water containing glycidol could show axonopathy and hippocampal nerve distortion (28). While in the hippocampal dentate gyrus, they found that Thbs2 could regulate the plasticity of neurons. Galectins control the important pathophysiological processes of the CNS. In addition, Lgals3 can promote the differentiation of oligodendrocyte, maintain the integrity of myelin, and promote the recovery of inflammatory demyelinating disease (32). Ctsd-knockout can lead to changes in the ultrastructure of myelin and metabolic disorders of cholesterol and the extreme absence of neurons in the brains of mice (33). In summary, the above proteins were found to be closely related to the pathophysiological processes of the nervous system, consistent with the results of the proteins that we screened.

ENPP3 is a member of the ectonucleotide pyro-phosphatase/phosphodiesterase family (E-NPPs). It has been reported that ENPP3 is present in almost all systems in the human body (34). Abnormal expression of ENPP3 can affect intracellular transduction pathways, leading to cellular dysfunction. A recent study has found the presence of ENPP1 and ENPP3 in rat podocytes and assessed their expression in rat podocytes cultured with 5 mM (normal glucose) or 30 mM glucose (high glucose) (35). In another study, the investigators examined the effect of endotoxin on nucleotide catabolism in the kidneys of mice by lipopolysaccharide (LPS) injection (36). The expression pattern of exogenous nucleotides showed that the level of Enpp3 mRNA was increased after LPS injection. Purine metabolic analysis by high-performance liquid chromatography assay confirmed this result. In our research, quantities of ENPP3 proteins were measured by Western blotting analysis, we found that the level of ENPP3 expression in SCs was significantly correlated with the peripheral nerve injury. Changes in protein abundance were consistent with proteomics data from SCs by Western blot analysis and protein quantification.

 In dendritic cells (DC), the GPNMB is a transmembrane protein that acts as a coinhibitory molecule strongly inhibiting the responses of T cell (37). Major histocompatibility complex class II (MHCII) molecules similarly expressed in DC subsets. In addition, MHCII was upregulated in cultured SCs and degenerated nerve tissue (38). Therefore, we speculated whether GPNMB and MHCII co-controlled the antigen presentation of DC cells. However, the specific mechanism and the immune regulation need further exploration. In addition, many studies reported a number of new markers for Schwann cells as early as 2012 (39), for example, TUBB3, ATG5, and NEFM. A study on spinal muscular atrophy (SMA) showed that ubiquitin-like modification 1 (Uba1) and ubiquitin-dependent pathways play an important role in maintaining Schwann cell homeostasis and provide important additional experimental evidence (40). The above proteins were also detected in this research, but the objective of this study was determining what kind of proteomics changes have occurred in Schwann cells before and after peripheral nerve injury, thus validating only four related proteins. Similarly, Lgals3 was detected in relation to the pathophysiological processes of the CNS or the peripheral nerves (41, 42), while the other two proteins were screened (Ctsd and Sptan1), and there was no clear literature to support its role in the nervous system. Thus, studies on the function of the proteins in nerve injury are urgently needed for further exploration.

Similar to previous studies, our research is based on the *in vitro* culture of SCs to study the changes in the microenvironment of SCs after peripheral nerve injury (43-45). Additionally, we explored the application of sciatic nerve pre-injury model, which is generally recognized, in Wistar rats (46). However, we first used iTRAQ technology to label potential biomarkers in SCs to explore the possible changes in SCs after peripheral nerve injury. Although important discoveries were revealed in this study, there are also some limitations. First, the sample size of this study needs to be further expanded, and the selected peripheral nerves should be more diverse. Second, the pathways selected in this study need to be further validated. The changes in SCs before and after nerve injury require further exploratory mechanisms. Finally, we just explored SCs *in vitro*; the transplantation of SCs in conjunction with other cells into animals is the next major task. 

Marking proteins based on iTRAQ technology is a popular topic in the current studies on protein labeling (47-49). We hope that this study further explored the changes in SCs in the peripheral environment after injury and provide a new approach for better clinical application of SCs.

## Conclusion

We used iTRAQ-Orbitrap LC-MS/MS technique and bioinformatics analysis to conduct a proteomics study to identify proteins that were differentially expressed between ASCs and NSCs. Based on our findings, GPNMB, ENPP3, Thbs2, and Lgals3 may play a key role in repair of SCs after peripheral nerve injury. Here, we report a new finding on SCs after nerve injury and warrants further studies in the future.

## References

[1] Birbeck GL, Meyer AC, Ogunniyi A (2015). Nervous system disorders across the life course in resource-limited settings. Nature.

[2] Martinez AR, Faber I, Nucci A, Appenzeller S, Franca MC (2017). Autoimmune neuropathies associated to rheumatic diseases. Autoimmun Rev.

[3] Huang H, Mao G, Chen L, Liu A (2015). Progress and challenges with clinical cell therapy in neurorestoratology. J Neurorestoratol.

[4] Gross G, Eshhar Z (2016). Therapeutic potential of T-cell chimeric antigen receptors (CARs) in cancer treatment: counteracting off-tumor toxicities for safe CAR T-cell therapy. Annu Rev Pharmacol Toxicol.

[5] Sarkar P, Rice CM, Scolding NJ (2017). Cell therapy for multiple sclerosis. CNS Drugs.

[6] Ansari S, Seagroves JT, Chen C, Shah K, Aghaloo T, Wu BM (2017). Dental and orofacial mesenchymal stem cells in craniofacial regeneration: the prosthodontist’s point of view. J Prosthet Dent.

[7] Boilly B, Faulkner S, Jobling P, Hondermarck H (2007). Nerve dependence: from regeneration to cancer. Cancer Cell.

[8] Zawadzka M, Rivers LE, Fancy SP, Zhao C, Tripathi R, Jamen F (2010). CNS-resident glial progenitor/stem cells produce Schwann cells as well as oligodendrocytes during repair of CNS demyelination. Cell Stem Cell.

[9] Koenig HL, Schumacher M, Ferzaz B, Thi AN, Ressouches A, Guennoun R (1995). Progesterone synthesis and myelin formation by Schwann cells. Science.

[10] Pearse DD, Pereira FC, Marcillo AE, Bates ML, Berrocal YA, Filbin MT (2004). cAMP and Schwann cells promote axonal growth and functional recovery after spinal cord injury. Nat Med.

[11] Park HW, Lim MJ, Jung H, Lee SP, Paik KS, Chang MS (2010). Human mesenchymal stem cell-derived Schwann cell-like cells exhibit neurotrophic effects, via distinct growth factor production, in a model of spinal cord injury. Glia.

[12] Carlson KB, Singh P, Feaster MM, Ramnarain A, Pavlides C, Chen ZL (2011). Mesenchymal stem cells facilitate axon sorting, myelination, and functional recovery in paralyzed mice deficient in Schwann cell-derived laminin. Glia.

[13] Lai BQ, Che MT, Du BL, Zeng X, Ma YH, Feng B (2016). Transplantation of tissue engineering neural network and formation of neuronal relay into the transected rat spinal cord. Biomaterials.

[14] Lavdas AA, Papastefanaki F, Thomaidou D, Matsas R (2011). Cell adhesion molecules in gene and cell therapy approaches for nervous system repair. Curr Gene Ther.

[15] Yi S, Tang X, Yu J, Liu J, Ding F, Gu X (2017). Microarray and qPCR analyses of wallerian degeneration in rat sciatic nerves. Front Cell Neurosci.

[16] Yu J, Gu X, Yi S (2016). Ingenuity pathway analysis of gene expression profiles in distal nerve stump following nerve injury: insights into wallerian degeneration. Front Cell Neurosci.

[17] Zhang L, Jia X, Jin JO, Lu H, Tan Z (2017). Recent 5-year findings and technological advances in the proteomic study of HIV-associated disorders. Genomics Proteomics Bioinformatics.

[18] Zhang P, Zhu S, Li Y, Zhao M, Liu M, Gao J (2016). Quantitative proteomics analysis to identify diffuse axonal injury biomarkers in rats using iTRAQ coupled LC–MS/MS. J Proteom.

[19] Chen J, Ge L, Liu A, Yuan Y, Ye J, Zhong J (2016). Identification of pathways related to FAF1/H pylori-associated gastric carcinogenesis through an integrated approach based on iTRAQ quantification and literature review. J Proteom.

[20] Mehrotra S, Goyal V (2013). Evaluation of designer crops for biosafety—A scientist’s perspective. Gene.

[21] Yang S, Pei Y, Zhao A (2017). iTRAQ-based proteomic analysis of porcine kidney epithelial PK15 cells infected with pseudorabies virus. Sci Rep.

[22] Woodhoo A, Alonso MBD, Droggiti A, Turmaine M, D’antonio M, Parkinson DB (2009). Notch controls embryonic Schwann cell differentiation, postnatal myelination and adult plasticity. Nat Neurosci.

[23] Keilhoff G, Fansa H, Schneider W, Wolf G (1999). In vivo predegeneration of peripheral nerves: an effective technique to obtain activated Schwann cells for nerve conduits. J Neurosci Methods.

[24] Zhou XH, Lin W, Ren YM, Liu S, Fan BY, Wei ZJ (2017). Comparison of DNA methylation in Schwann cells before and after peripheral nerve injury in rats. BioMed Res Int.

[25] Hu X, Li N, Wu L, Li C, Li C, Zhang L (2015). Quantitative iTRAQ-based proteomic analysis of phosphoproteins and ABA-regulated phosphoproteins in maize leaves under osmotic stress. Sci Rep.

[26] Ma J, Yao Y, Wang P, Liu Y, Zhao L, Li Z (2014). MiR-152 functions as a tumor suppressor in glioblastoma stem cells by targeting Kruppel-like factor 4. Cancer Lett.

[27] Burnside MN, Pyatt RE, Hughes A, Baker PB, Pierson CR (2015). Complex brain malformations associated with chromosome 6q27 gain that includes THBS2, which encodes thrombospondin 2, an astrocyte-derived protein of the extracellular matrix. Pediatr Dev Pathol.

[28] Akane H, Saito F, Shiraki A, Imatanaka N, Akahori Y, Itahashi M (2014). Gene expression profile of brain regions reflecting aberrations in nervous system development targeting the process of neurite extension of rat offspring exposed developmentally to glycidol. J Appl Toxicol.

[29] Huber RJ (2016). Using the social amoeba Dictyostelium to study the functions of proteins linked to neuronal ceroid lipofuscinosis. Int J Biomed Sci.

[30] Nonoda Y, Saito Y, Nagai S, Sasaki M, Iwasaki T, Matsumoto N (2013). Progressive diffuse brain atrophy in West syndrome with marked hypomyelination due to SPTAN1 gene mutation. Brain Dev.

[31] Cáceres M, Suwyn C, Maddox M, Thomas JW, Preuss TM (2006). Increased cortical expression of two synaptogenic thrombospondins in human brain evolution. Cereb Cortex.

[32] Pasquini LA, Millet V, Hoyos HC, Giannoni JP, Croci DO, Marder M (2011). Galectin-3 drives oligodendrocyte differentiation to control myelin integrity and function. Cell Death Differ.

[33] Mutka AL, Haapanen A, Käkelä R, Lindfors M, Wright AK, Inkinen T (2010). Murine cathepsin D deficiency is associated with dysmyelination/myelin disruption and accumulation of cholesteryl esters in the brain. J Neurochem.

[34] Bollen M, Gijsbers R, Ceulemans H, Stalmans W, Stefan C (2000). Nucleotide pyrophosphatases/phosphodiesterases on the move. Crit Rev Biochem Mol Biol.

[35] Jankowski M, Piwkowska A, Rogacka D, Audzeyenka I, Janaszak-Jasiecka A, Angielski S (2011). Expression of membrane-bound NPP-type ecto-phosphodiesterases in rat podocytes cultured at normal and high glucose concentrations. Biochem biophys res commun.

[36] Vuaden FC, Savio LE, Ramos DB, Casali EA, Bogo MR, Bonan CD (2012). Endotoxin-induced effects on nucleotide catabolism in mouse kidney. Eur J Pharmacol.

[37] Gutknecht M, Geiger J, Joas S, Dorfel D, Salih HR, Muller MR (2015). The transcription factor MITF is a critical regulator of GPNMB expression in dendritic cells. Cell commun signal.

[38] Weiss T, Taschner-Mandl S, Bileck A, Slany A, Kromp F, Rifatbegovic F (2016). Proteomics and transcriptomics of peripheral nerve tissue and cells unravel new aspects of the human Schwann cell repair phenotype. Glia.

[39] Shen M, Ji Y, Zhang S, Shi H, Chen G, Gu X (2012). A proteome map of primary cultured rat Schwann cells. J Proteome Sci.

[40] Aghamaleky Sarvestany A, Hunter G, Tavendale A, Lamont DJ, Llavero Hurtado M, Graham LC (2014). Label-free quantitative proteomic profiling identifies disruption of ubiquitin homeostasis as a key driver of Schwann cell defects in spinal muscular atrophy. J Proteome Res.

[41] Kawahara K, Hirata H, Ohbuchi K, Nishi K, Maeda A, Kuniyasu A (2016). The novel monoclonal antibody 9F5 reveals expression of a fragment of GPNMB/osteoactivin processed by furin-like protease(s) in a subpopulation of microglia in neonatal rat brain. Glia.

[42] Coughlin L, Morrison RS, Horner PJ, Inman DM (2015). Mitochondrial morphology differences and mitophagy deficit in murine glaucomatous optic nerve. Invest Ophthalmol Vis Sci.

[43] Du J, Liu J, Yao S, Mao H, Peng J, Sun X (2017). Prompt peripheral nerve regeneration induced by a hierarchically aligned fibrin nanofiber hydrogel. Acta Biomater.

[44] Liao CP, Pradhan S, Chen Z, Patel AJ, Booker RC, Le LQ (2016). The role of nerve microenvironment for neurofibroma development. Oncotarget.

[45] Dey I, Midha N, Singh G, Forsyth A, Walsh SK, Singh B (2013). Diabetic Schwann cells suffer from nerve growth factor and neurotrophin-3 underproduction and poor associability with axons. Glia.

[46] Civi S, Emmez G, Dere UA, Borcek AO, Emmez H (2016). Effects of quercetin on chronic constriction nerve injury in an experimental rat model. Acta Neurochir.

[47] Wu Z, Ding N, Yu M, Wang K, Luo S, Zou W (2016). Identification of potential biomarkers for rhegmatogenous retinal detachment associated with choroidal detachment by vitreous iTRAQ-based proteomic profiling. Int J Mol Sci.

[48] Subbannayya Y, Mir SA, Renuse S, Manda SS, Pinto SM, Puttamallesh VN (2015). Identification of differentially expressed serum proteins in gastric adenocarcinoma. J Proteom.

[49] Wang Y, Liu H, Liang D, Huang Y, Zeng Y, Xing X (2017). Reveal the molecular signatures of hepatocellular carcinoma with different sizes by iTRAQ based quantitative proteomics. J Proteom.

